# Improper Use of Antipyretics

**Published:** 1891-04

**Authors:** 


THE IMPROPER USE OF ANTIPYRETIS.—Every physician should read Dr. Keown's paper in this issue. While the Doctor would not appear as an alarmist, he very properly points out the necessity of great care and discrimination in the use of the coal tar products, and shows by a rather extraordinary experience and observation, that improperly or injudiciously used, they, or some of them, at least, are very *dangerous*. It is a valuable paper.

